# Visceral leishmaniasis due to *Leishmania infantum*with renal involvement in HIV-infected patients

**DOI:** 10.1186/s12879-014-0561-9

**Published:** 2014-10-30

**Authors:** Matteo Vassallo, Olivier Moranne, Damien Ambrosetti, Pierre-Yves Jeandel, Christelle Pomares, Elisabeth Cassuto, Annick Boscagli, Guillaume Giraud, Nathalie Montagne, Chiara Dentone, Ilaria Demacina, Barbara Villaggio, Giovanni Secondo, Giuseppe Ferrea, Corinne Passeron, Laurence Saudes, Regis Kaphan, Pierre Marty, Eric Rosenthal

**Affiliations:** Department of Internal Medicine, Cannes General Hospital, Cannes, France; Department of Nephrology, Dialysis and Transplantation, Nice University Hospital, Nice, France; Department of Public Health, Nice University Hospital, Nice, France; Laboratory of Human Motricity, Education and Health (LAMHESS), University of Nice Sophia-Antipolis, Nice, France; Department of Histopathology, Nice University Hospital, Nice, France; Department of Internal Medicine, Nice University Hospital, Nice, France; Parasitology and Mycology, Nice University Hospital and Inserm U 1065, Nice-Sophia Antipolis University, Equipe 6, Centre Méditerranéen de Médicine Moléculaire, Nice, France; Department of Infectious Diseases, Sanremo General Hospital, Sanremo, Italy; Department of Histopathology, Genoa University Hospital, Genoa, Italy; Department of Nephrology, Cannes General Hospital, Cannes, France

## Abstract

**Background:**

We describe histological, clinical findings and outcomes of renal involvement during *Leishmania infantum* infection in four HIV-infected patients in South France and North Italy hospital settings.

**Cases presentation:**

Four HIV-infected Caucasian patients (age 24-49) performed renal biopsy during episodes of visceral leishmaniasis. They presented severe immunosuppression, frequent relapses of visceral leishmaniasis during a follow-up period of several years and partial or complete recovery of renal function after anti-parasitic treatment. Main clinical presentations were nephrotic or nephritic syndrome and/or acute renal failure secondary to membranoproliferative type III glomerulonephritis or acute interstitial nephritis. Clinical outcome was poor, probably as a consequence of insufficient immuno-virological control of the HIV infection.

**Conclusions:**

Our findings suggest that the main histological findings in case of renal involvement due to *Leishmania infantum* infection in HIV-infected patients are type III MPGN and acute interstitial nephritis, with a histological specificity similar to that observed in canine leishmaniasis. Poor immune status in HIV-infected patients, altering the capacity for parasite clearance, and prolonged course of chronic active VL in this population may lead to the development of specific renal lesions.

**Electronic supplementary material:**

The online version of this article (doi:10.1186/s12879-014-0561-9) contains supplementary material, which is available to authorized users.

## Background

Leishmaniasis due to *Leishmania infantum* is a vector-borne disease which is endemic in Southern Europe [1]-[5]. In humans, *Leishmania* infection gives rise to a range of presentations (mainly visceral, cutaneous and mucosal) depending on the species involved and the effectiveness of the patient’s immune response to the parasite [2],[6],[7]. Visceral leishmaniasis (VL) is present in 61 countries across 4 continents where 200 million people are exposed. Approximately 0.2 to 0.4 million cases of VL and 0.7 to 1.2 million cases of cutaneous leishmaniasis occur each year, with 5 countries (India, Nepal, Bangladesh, Sudan, Brazil) accounting for 90% of these, whereas the condition remains sporadic in Southern Europe [1]-[4]. In immunosuppressed individuals, including HIV-infected patients, the clinical presentation of VL can be atypical [8], easily misdiagnosed or mistaken as a flare-up of the underlying disease. Before the introduction of combination antiretroviral therapy (cART), the incidence of VL was higher in HIV-infected patients than in immunocompetent subjects, with higher rates of relapse and mortality [2]. In the cART era, the incidence of VL in the HIV-infected population has dramatically decreased [9]-[13]. Moreover, with the longer life expectancy of HIV-infected individuals, new clinical features of VL have been reported, such as active chronic VL, characterized by relapses over a period of several years and continuous circulation of the parasite in the bloodstream [14].

Renal involvement in HIV-infected patients with VL is uncommon and few cases have been reported [15]-[19]. Patients displayed various patterns of clinical and histological presentation: isolated proteinuria or hematuria, nephrotic syndrome (proteinuria >2.5 g/day, hypoalbuminemia and generalized oedema), nephritic syndrome (glomerular proteinuria & hematuria, reduced glomerular filtration rate with varying degrees of azotemia, oliguria and hypertension) secondary to membrano-proliferative glomerulonephritis (MPGN) or amyloidosis, and acute kidney injury (AKI) secondary to interstitial nephritis [15]-[22].

We present a series of four cases of VL due to *Leishmania infantum* with renal involvement in HIV-infected patients, including histological findings and clinical outcome. Patients were hospitalized between 1996 and 2012 in Infectious Diseases Hospital departments in the Alpes-Maritimes, France and in Liguria, Italy, where *Leishmania infantum* infection is endemic.

## Cases presentation

### Case report n° 1

A 40-year-old HIV-infected male patient, born and residing in the Nice area (Alpes-Maritimes, South-Eastern France), was admitted to hospital in June 2011 for persistent diarrhoea and weight loss (5 kgs). He had contracted HIV-infection in 1996, and his previous medical history included VL in 2000, (with clinical relapses in 2005 and 2009 but no renal involvement), high blood pressure, cerebral toxoplasmosis and Kaposi’s sarcoma in 2003, and pneumocystis pneumonia in 2006. His antiretroviral regimen included etravirine, darunavir and ritonavir. The latest CD4 cell count was 114 cells/mm^3^ (nadir: 46 cells/mm^3^) and HIV viral load was 162 copies/ml. Compliance with cART had frequently been sub-optimal. He presented with nephritic syndrome, urine dipstick analysis revealing proteinuria and hematuria, but no leukocyturia. Blood pressure was 150/100 mmHg, with peripheral œdema. All kidney tissue samples were obtained via needle biopsy. Light microscopy examination showed diffuse hyper-cellularity with thickening of the glomerular basement membrane, and immunofluorescence microscopy revealed IgG, IgM, C3 and C1q mesangial and endo-membranous deposits . According to the World Health Organisation (WHO) classification, the histological findings were typical of type III membrano-proliferative glomerulonephritis (MPGN).

Diagnosis of VL was based on molecular methods using *Leishmania infantum* polymerase chain reaction (PCR) (Taqman technology; Light Cycler target: kinetoplast mini-circle DNA) on peripheral blood and other biological samples, i.e. bone marrow, urine and kidney [23]. In this patient, qualitative PCR was positive in both urine and peripheral blood. VL relapse was suspected and liposomal Amphotericine B (l-Ampho-B), 3 mg/kg daily, initially scheduled for 10 days, was prematurely discontinued due to deteriorating renal function and suspected drug-associated tubulopathy. The patient’s condition gradually improved and secondary VL prophylaxis consisting in monthly intravenous pentamidine infusions was initiated. During follow-up, creatinine levels remained stable and viro-immunological parameters improved. The patient was re-admitted in October 2013 for another relapse of VL. Poor cART compliance was suspected. The CD4 T-cell count and plasma HIV-RNA level were 79 cells/mm^3^ and 1,500 copies/ml, respectively. PCR was positive for *Leishmania infantum* with 992 parasites/ml in plasma and 322 parasites/10^6^ cells in bone marrow. Treatment with l-Ampho-B was started, adapting dosage to renal function; fever resolved, parasitemia dropped to 312 parasites/ml and the estimated glomerular filtration rate (eGFR) remained stable. Antiretroviral treatment was switched from a twice-daily to a once-daily regimen, in order to improve compliance. The patient was discharged and continued secondary VL prophylaxis as an outpatient with L-Ampho-B.

Table 1 summarizes baseline characteristics, renal impairment and clinical outcome for all four patients, while the main histological findings are described in Table 2.

**Table 1:** **Description of cases of VL with renal injury and outcome**

	Case 1	Case 2	Case 3	Case 4
Clinical characteristics				
**Age**	40	35	24	49
**Years since HIV contamination**	15	11	24	16
**Years since diagnosis of visceral Leishmaniosis**	11	9	2	19
**Renal syndrome**	Nephritic syndrome	Nephrotic syndrome	AKI	Nephrotic syndrome
**Biology**				
***Leishmania*** **quantitative PCR test (parasites/ml)**	6225 (blood)	1700000 (kidney)	Not available	11570 (blood)
**C4 concentration (normal range 0.14-0.33 g/l)**	0.12	Not available	0.14	0.2
**Cryoglobulin**	Positive	Not available	Positive	Positive type 2
**HCV serology**	Negative	Negative	Negative	Positive
**HCV viral load in blood (qualitative)**	-	-	-	Negative
**Serum albumin (g/l) at time of diagnosis or during follow-up**	25	22/14	-	20/24
**Plasma hyper-gammaglobulinemia**	Yes	Unknown	Yes	Yes
**Platelets >150000 giga/L at time of renal biopsy**	Yes	Yes	Yes	Yes
**Severe clottingdysfunction**	No	No	No	No
**Hepatic function**	Normal	Normal	Normal	Normal
**HIV viral load (copies/ml)**	162	41000	2000	< 40
**CD4 T-cell count (cells/mm** ^**3**^ **)**	114	12	70	84
**CD4 T-cell nadir (cells/mm** ^**3**^ **)**	46	12	50	84
**Plasma creatinine concentrations (year, [μmol/l]) diagnosis/follow-up**	143/152	2006 (100)/2007 (265)/2008(488)	154 / 99	55 / 60
**CKD-EPI (ml/min/1.73 m** ^**2**^ **) diagnosis/follow-up**	48/47	105 / 90 / 52		69
**Urinalysis**				
**Daily proteinuria (g/day) Diagnosis/follow-up**	2.1	1.5/2.0/3.0	0.3 / 0.5	2.0 / 0.5
**Hematuria/Leukocyturia**	+/-	+/-	-/+	-/-
**Kidney histological findings**	MPGN III	MPGN III and Interstitial nephropathy	Interstitial nephropathy	MPGN III and interstitial nephropathy
**Treatment and outcome**				
**VL treatment regimen**	l-Amph-B, Pentamidine	l-Amph-B, Pentamidine,	l-Amph-B	l-Amph-B, Miltéfosime
		Miltefosine		
**Renal outcome**	Improved	Not improved	Improved	Improved but worsened at clinical relapse
**Patient outcome**	Partial recovery	Died	Partial recovery	Died

VL: Visceral Leishmaniasis.

PCR: Polymerase Chain Reaction.

CKD-EPI: Chronic Kidney Disease Epidemiology Collaboration Equation.

l-Amph-B: liposomal Amphotericine B.

**Table 2:** **Description of kidney histo-pathological diagnosis**

Patient	Case 1	Case 2	Case 3	Case 4
Renal histology	MPGN type III	MPGN type III	Acute interstitial nephritis	MPGN type III
**Light microscopy**	Diffuse hypercellularity and thickening of glomerular basement membrane.	Subendothelial and subepithelial deposits with mesangial interposition associated with basement membrane spikes	Acute interstitial nephritis: edema associated with interstitial lymphoplasmocytic infiltration in addition to mild tubulointerstitial fibrosis.	Subendothelial and subepithelial deposits and mesangial interposition associated with basement membrane spikes.
		Free *Leishmania* in a capillary lumen	Tubules are invaded by lymphocytes and areas of necrosis are visualized.	Interstitial granuloma. Positive PCR for *Leishmania infantum* in urine and in renal biopsy
		Positive PCR for *Leishmania infantum* in the renal biopsy	Glomeruli are uninvolved. Free *Leishmania* in a capillary lumen	
**Immunofluorescence**	Mesangial and endomembranous deposits of IgG, IgM, C3 and C1q	Fine granular deposition of IgG and C3 in the mesangium and along the peripheral capillary loops	Deposits of IgG and IgM with a mesangial pattern	Mesangial and endomembranous deposits of IgG and C3

MPGN: Membrano-proliferative glomeruolonephritis.

PCR: Polymerase Chain Reaction.

### Case report n° 2

A 35-year-old male patient, HIV-infected since 1997, born and residing in the Nice area (Alpes-Maritimes, South-Eastern France), was first admitted to hospital in 1998 with VL. He was treated with l-Ampho-B and symptoms improved. During the following years, the patient had several relapses of VL despite various secondary prophylaxis regimens, including l-ampho-B, pentamidine and miltefosine. In 2004 he was re-admitted with suspected clinical relapse of VL. His CD4 cell count was 20 cells/mm^3^ while HIV viral load exceeded 100,000 copies/ml. *Leishmania infantum* parasites were found both in colon biopsy samples and in peripheral blood. Moreover, liver biopsy showed large macrophages containing *Leishmania infantum.* An additional course of l-Ampho-B resulted in clinical recovery and was followed by maintenance therapy. The eGFR was normal. Compliance with cART was poor and the CD4 T-cell count remained below 200 cells/mm^3^.

In 2006 the patient presented with nephrotic syndrome. C4 and C3 concentrations were normal, while HIV viral load and CD4 T-cell count were 41,000 copies/ml and 12 cells/mm^3^, respectively. *Leishmania* amastigotes were observed on a blood smear. A renewed course of l-ampho-B resulted in remission of the nephrotic syndrome.

**Figure 1:**
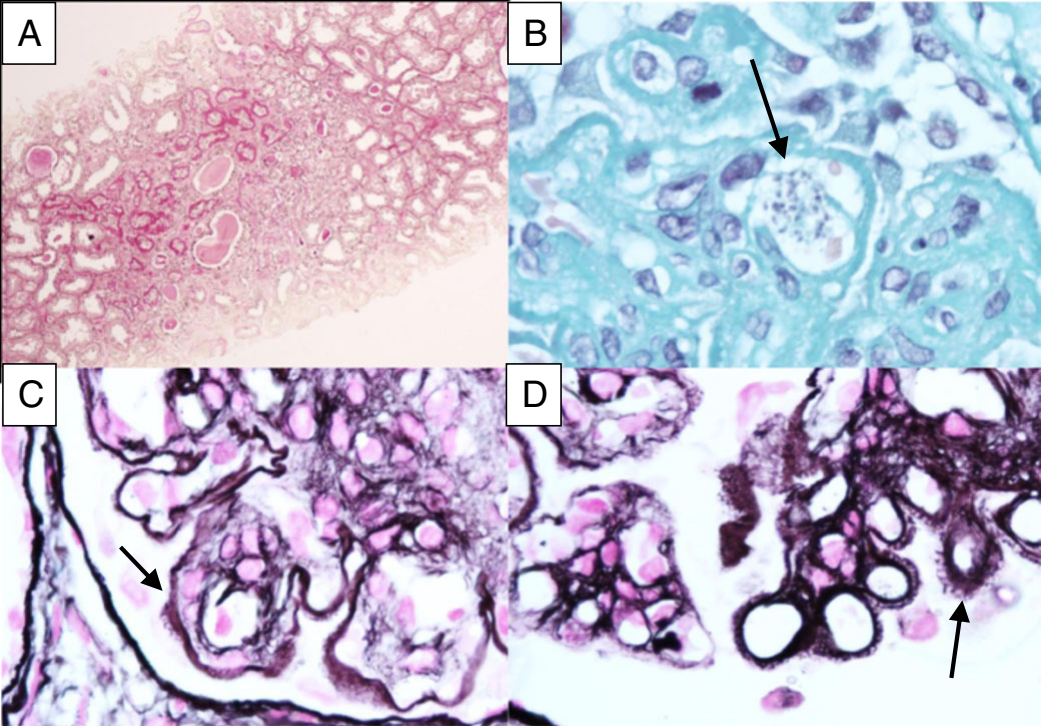
**Main histological findings in patient 2, who had type III membrano-proliferative glomerulonephritis (MPGN). A**. Cortical kidney inflammation (PAS x1000). **B**. Clusters of circulating *Leishmania* free parasytes in capillary (arrow) (Trichrome stain x1000). **C**. Prominent double contour formations (arrow) (Silver stain x1000). **D**. Basement membrane spikes (Silver stain x1000).

In 2007, the patient was admitted again with œdema, a weight gain of 4 kgs and dyspnœa. He presented this time with nephritic syndrome and acute kidney injury. Complement activity was normal. Gastroscopy and colonoscopy revealed several parasites in the stomach, duodenum and colon. Qualitative *Leishmania infantum* PCR on blood and gut samples was positive. Histological examination of kidney tissue showed type III MPGN with free *Leishmania* parasites in a capillary lumen (Figure 1A,B,C,D). Moreover, *Leishmania infantum* PCR was positive on the biopsy sample.

Compliance with antiretroviral treatment remained poor and in 2008 the patient was admitted to hospital for the fourth time due to renewed relapse with diarrhœa, weight loss and œdema. His HIV-RNA and CD4 T-cell count were 74,000 copies/ml and 200 cells/mm^3^, respectively. The patient died due to renal failure despite anti-parasitic treatment with l-Ampho-B.

### Case report n° 3

A 24 year-old female patient, born in Italy and a resident of the Sanremo area (Liguria, North-Western Italy), HIV-infected via maternal-fœtal transmission, was admitted to hospital in 2012 for weight loss and AKI.

**Figure 2:**
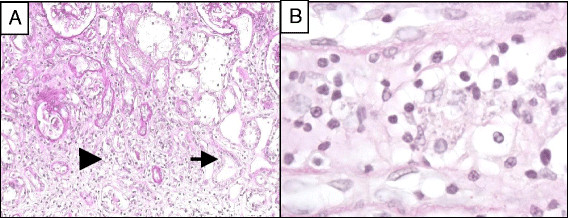
**Main histological results in patient 3, who presented acute interstitial nephritis without glomerular involvement. A**. Acute interstitial nephritis: oedema associated with an infiltration of interstitial lymphoplasmacytoid cells (Arrowhead). Tubules are invaded by lymphocytes (Arrow). Glomeruli are uninvolved. (PAS x200). **B**. Free *Leishmania* in interstitial tissue (PAS x1000).

At the time of admission, her cART regimen consisted in etravine, raltegravir and boosted darunavir. The CD4 T-cell count was low (70 cells/mm^3^) and plasma HIV-RNA numbered2,000 copies/ml. Previous personal medical history included pneumocystis pneumonia, œsophageal candidiasis, cytomegalovirus retinitis and, more recently, two episodes of VL over the last two years. Both episodes were treated with l-Ampho-B followed by secondary prophylaxis. Several antiretroviral regimens had been administered, but compliance had never been satisfactory, viral load had been undetectable during only a few months and the CD4 T-cell count had consistently remained below 200 cells/mm^3^, with a nadir of 50 cells/mm^3^. Gastric and lymph node biopsy revealed *Leishmania infantum*, and a renal needle biopsy was performed to investigate AKI associated with proteinuria. Light microscopy examination showed interstitial nephritis with free Leishmania in interstitial tissue while no involvement of the glomeruli was observed. Immunofluorescence microscopy revealed a mesangial pattern of IgG and IgM deposits (Figure 2A,B)

Treatment with l-Ampho-B was started and resulted in improved eGFR. Antiretroviral treatment was modified according to genotyping results. The patient was discharged and secondary prophylaxis with l-Ampho-B was continued. A recent outpatient follow-up visit showed stable renal function, normal urinary sediment microscopy and proteinuria of 0.53 g/24 hrs. Despite the persistent clinical relapse-free period, PCR remained positive on blood (736 parasites/ml) with a CD4 T-cell count below 200 cells/mm^3^ while the HIV viral load did not exceed50 copies/ml.

### Case report n° 4

A 49-year-old male patient, born and residing in the Nice area (Alpes-Maritimes, South-Eastern France), HIV and hepatitis C (HCV) co-infected since the age of 16, was admitted to hospital in 2011 for weight loss, œdema and nephrotic syndrome without hematuria. The eGFR was normal. VL had been previously diagnosed in 1991 and treated, resulting in symptom resolution.

Laboratory tests upon admission showed undetectable HCV and HIV viral loads, and a CD4 T-cell count of 84 cells/mm^3^. The antiretroviral regimen included abacavir, lamivudine and lopinavir/ritonavir.

**Figure 3:**
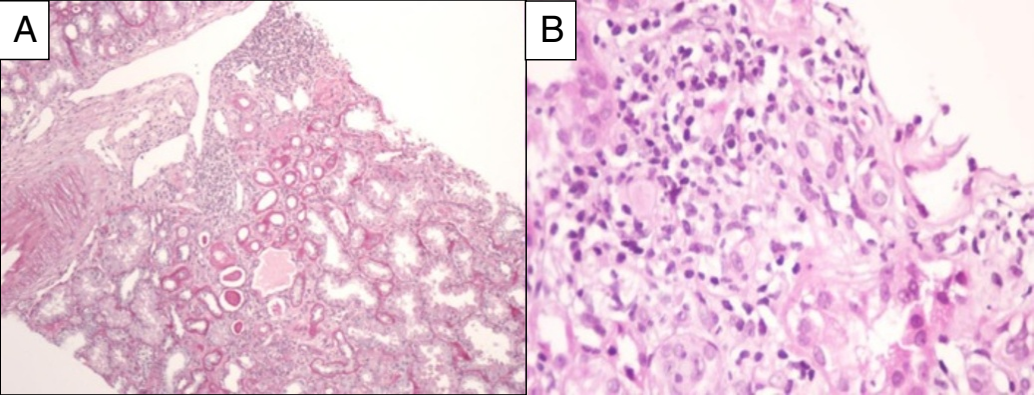
**Description of renal biopsy results for patient 4, who presented type III MPGN. A**. Inflammation within the cortical kidney (PAS x100). **B**. Giant cell granuloma. (HE stain x 400).

Antiparasitic treatment with miltefosine was initiated, resulting in clinical and biological improvement. *Leishmania infantum* PCR on blood was negative, but was positive on urine. Kidney histological findings showed type III MPGN and interstitial granuloma. with positive *Leishmania infantum* PCR on kidney tissue (Figure 3A,B). Although HCV co-infection may also cause renal injury, the undetectable HCV viral load and positive *Leishmania infantum* PCR suggested that MPGN was more likely to be linked to VL than to HCV. One year later, the patient was re-admitted due to clinical relapse of VL. HIV-RNA on admission was 1,700 copies/ml and CD4 T-cells numbered 78/mm^3^. *Leishmania infantum* PCR on blood was positive (11,570 parasites/ml). In spite of treatment with l-Ampho-B, the patient died a few days after admission due to severe intestinal bleeding.

## Discussion

We describe four cases of VL due to *Leishmania infantum* in HIV-infected patients in the cART era, with specific PCR or microscopic proof of renal involvement.

All patients had severe immunosuppression, and had presented with recurring episodes of clinical relapse of VL over a follow-up period of several years. Moreover, they had partial or complete recovery of renal function after anti-parasitic treatment associated with better control of HIV infection. The main clinical presentations included nephrotic or nephritic syndrome secondary to type III MPGN or acute kidney injury secondary to interstitial nephritis.

According to the WHO classification, based on ultrastructural alterations in the glomerular basement membrane, MPGN is subdivided into 3 categories: type I (sub-endothelial deposits), type II (intra-membranous dense deposits) and type III, where deposits are found in the sub-endothelial and mesangial region, similarly to type I, but, in addition, with numerous sub-epithelial deposits [24]. Histological findings reported in our series are in line with sporadic cases described in humans and in studies on dogs, where type III MPGN was the most commonpattern [15]-[22],[25],[26]. Interestingly, free *Leishmania* were observed inside the capillary lumen of two patients.

MPGN during VL is probably the result of immune complex deposition occurring during chronic infection [15],[24],[27]. However, other mechanisms of injury have been suggested in dogs: indeed, there is growing evidence that T-cell migration into the glomeruli and adhesion molecules play a fundamental role in the pathogenesis of certain immunologically-mediated cases of glomerulonephritis [27]. Costa *et al.*[27] found considerably higher numbers of CD4 T-cells in dogs with VL compared to uninfected animals, with no relevant associated immune complex deposition. The number of T-cells was correlated to the quantity of *Leishmania* antigen in phagocytic cells, suggesting that the parasite itself could directly drive the inflammatory infiltrate in the glomeruli. Enhanced expression of adhesion molecules, such as P-selectin and ICAM-1, has also been found in the mesangium and glomerular capillaries of animals and humans with VL, thus suggesting that newly migrated platelets could play a role in the pathogenesis of renal injury. Finally, diminished apoptosis may be another possible mechanism contributing to the persistence and progression of glomerular hyper-cellularity [27].

Acute interstitial nephritis was the other presentation of renal involvement secondary to *Leishmania infantum* infection reported in this series. This is probably the result of parasitic dissemination, as reported by other authors in a human kidney transplant recipient [19] and in dogs [25],[26]. Tubular necrosis could be secondary to ischemia due to small vessel obliteration by *Leishmania spp.*, while haemolysis and necrotizing tubulitis could be a consequence of parasitic invasion and inflammatory response [28],[29].

The correlation between immunosuppression and clinical relapse of VL has been suggested by Bourgeois *et al.*, who showed that the persistence of CD4 T-cell counts <100 cells/mm^3^ is one of the main risk factors for relapse [30]. Since the advent of cART, features of HIV/*Leishmania spp.* coinfection include a chronic course of parasitic disease with latent parasitemia and a high rate of relapse. Bourgeois *et al.*[14] defined the condition as “active chronic VL”, where patients have long-term persistence of circulating *Leishmania* species with alternating symptomatic and asymptomatic phases. As showed by Kubar *et al*. [31], patients with relapsing VL generally have lower CD4 T-cell counts than HIV-infected patients with primary VL, suggesting that in addition to the T-cell-mediated response, essential at the time of primary *Leishmania* infection, supplementary mechanisms might operate in case of chronic infection.

Patients described in this series received various antiparasitic agents including l-Ampho-B, pentamidine and miltefosine. They all responded, at least partially, but they sufferedseveral relapses despite secondary prophylaxis. Data regarding secondary prophylaxis of VL in HIV-infected patients are scarce, explaining the absence of guidelines favouring a particular regimen [32]. There is no consensus on the management of active chronic VL. It is currently suggested that parasite detection by PCR, together with CD4 T-cell count, may guide decisions for prolonging or stopping prophylaxis in HIV-infected patients [33]-[36]. Bourgeois *et al.* suggest that secondary prophylaxis could be discontinued in patients taking cART when either 1) PCR remains negative for at least 6 months and CD4 T-cell count is >200 cells/mm^3^ or 2) CD4 T-cell count is <200 cells/mm^3^ but PCR remains negative for at least 18 months [30].

This case series showed that the clinical outcome of VL and renal injury is associated with viro-immunological control of HIV infection, suggesting that immune restoration plays a pivotal role in preventing relapse. In this case series, suboptimal compliance with antiretroviral treatment was probably the main cause of poor immune recovery, as proven by frequent episodes of viral replication during follow-up. However, VL itself might play a role: Leishmania co-infection could enhance viral replication and immune activation, thus contributing to CD4 T-cell depletion and increasing the risk of reactivation of latent infections [37],[38]. Thus, even patients with successful antiretroviral treatment could be at risk for *Leishmania* re-activation.

Finally, in this series, patients presented with active chronic VL progressing over a protracted course, ranging from 2 to 19 years (median 8 yrs). In mixed cryoglobulinemia due to hepatitis C virus, another condition mediated by immune complex deposition, vasculitis-related symptoms, including renal involvement, are associated with longer duration of HCV infection [39]. Similarly, in active chronic VL, prolonged infection may be required to develop renal disease.

## Conclusions

Our results suggest that the main histological findings in case of renal involvement due to *Leishmania infantum* infection in HIV-infected patients are type III MPGN and acute interstitial nephritis, with specific histological features similar to those observed in canine leishmaniasis. Poor immune status in HIV-infected patients, altering the capacity for parasite clearance, and prolonged course of chronic active VL in this population may lead to the development of these specific renal lesions. These patients should thus be regularly screened for Leishmania infantum infection and their renal function closely monitored.

## Consent

Written informed consent was obtained from patients for publication of this Case report and any accompanying images. A copy of the written consent is available for review by the Editor of this journal.

## Authors’ original submitted files for images

Below are the links to the authors’ original submitted files for images. Authors’ original file for figure 1 Authors’ original file for figure 2 Authors’ original file for figure 3
