# Supplementary figures and images for: Visceral leishmaniasis due to Leishmania infantumwith renal involvement in HIV-infected patients
Source: BMC Infect Dis. 2014 Oct 30;14:561. doi: 10.1186/s12879-014-0561-9 (PMC4216653; doi:10.1186/s12879-014-0561-9)

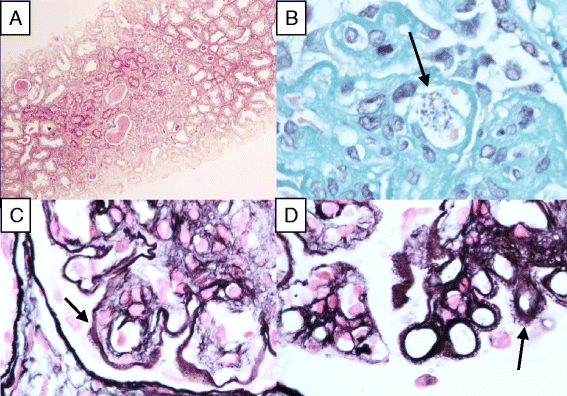

Supplement: Supplementary file 1 — Authors’ original file for figure 1 [file 12879_2014_561_MOESM1_ESM.gif]

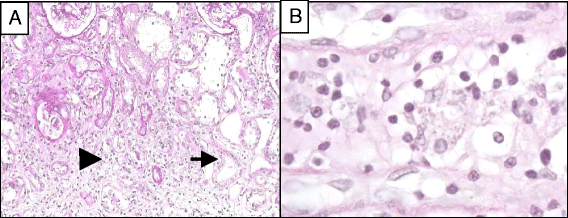

Supplement: Supplementary file 2 — Authors’ original file for figure 2 [file 12879_2014_561_MOESM2_ESM.gif]

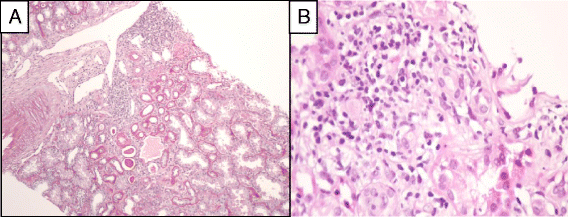

Supplement: Supplementary file 3 — Authors’ original file for figure 3 [file 12879_2014_561_MOESM3_ESM.gif]
